# The construction of confidence in a perceptual decision

**DOI:** 10.3389/fnint.2012.00079

**Published:** 2012-09-21

**Authors:** Ariel Zylberberg, Pablo Barttfeld, Mariano Sigman

**Affiliations:** ^1^Laboratory of Integrative Neuroscience, Physics Department, FCEyN UBA and IFIBAConicet, Buenos Aires, Argentina; ^2^Instituto de Ingeniería Biomédica, Facultad de Ingeniería, Universidad de Buenos AiresBuenos Aires, Argentina; ^3^Department of Vision and Cognition, Netherlands Institute for Neuroscience, An Institute of the Royal Netherlands Academy of Arts and SciencesAmsterdam, Netherlands

**Keywords:** perceptual decision-making, confidence, metacognition, psychophysical reverse-correlation, classification images, accumulation models of decision-making

## Abstract

Decision-making involves the selection of one out of many possible courses of action. A decision may bear on other decisions, as when humans seek a second medical opinion before undergoing a risky surgical intervention. These “meta-decisions” are mediated by confidence judgments—the degree to which decision-makers consider that a choice is likely to be correct. We studied how subjective confidence is constructed from noisy sensory evidence. The psychophysical kernels used to convert sensory information into choice and confidence decisions were precisely reconstructed measuring the impact of small fluctuations in sensory input. This is shown in two independent experiments in which human participants made a decision about the direction of motion of a set of randomly moving dots, or compared the brightness of a group of fluctuating bars, followed by a confidence report. The results of both experiments converged to show that: (1) confidence was influenced by evidence during a short window of time at the initial moments of the decision, and (2) confidence was influenced by evidence for the selected choice but was virtually blind to evidence for the non-selected choice. Our findings challenge classical models of subjective confidence—which posit that the difference of evidence in favor of each choice is the seed of the confidence signal.

## Introduction

In perceptual decisions, reaction times and accuracy distributions can be explained with great quantitative detail by models in which sensory evidence is accumulated to a threshold (Gold and Shadlen, 2007; Ratcliff and McKoon, 2008). A tempting hypothesis is that confidence reflects the accumulated signal at the moment of choice (Vickers, 1979; Moreno-Bote, 2010). Supporting this view, neurophysiological signals indexing choice confidence have been found in the same neurons encoding the accumulation process (Kiani and Shadlen, 2009).

However, other studies have signaled dissociations between confidence and accuracy whereby accurate responses were systematically observed at low confidence and erred responses at very high confidence (Graziano and Sigman, 2009). Transcranial magnetic stimulation (TMS) applied to the visual cortex can decrease accuracy but increase decision confidence (Rahnev et al., 2011), and lesion and TMS inactivation of dorso-lateral prefrontal cortex can impair human ability to estimate choice confidence without affecting performance (Del Cul et al., 2009; Rounis et al., 2010). The role of prefrontal cortex actively linking objective performance to subjective beliefs is also implied by correlations between a subject's meta-cognitive ability and gray matter volume in the anterior prefrontal cortex (Fleming et al., 2010).

Hence, there is contradictory evidence on whether confidence indexes a faithful analog measure of the accumulation process or, conversely, a partially distorted estimation of the decision signal. Here we address this issue quantitatively, comparing the empirical kernels (Eckstein and Ahumada, 2002) used to construct choice and confidence in simple decisions made on fluctuating motion (Kiani and Shadlen, 2009) or luminance (Neri and Heeger, 2002) signals.

## Results

### Experiment 1: choice and confidence in motion discrimination

**Figure 1:**
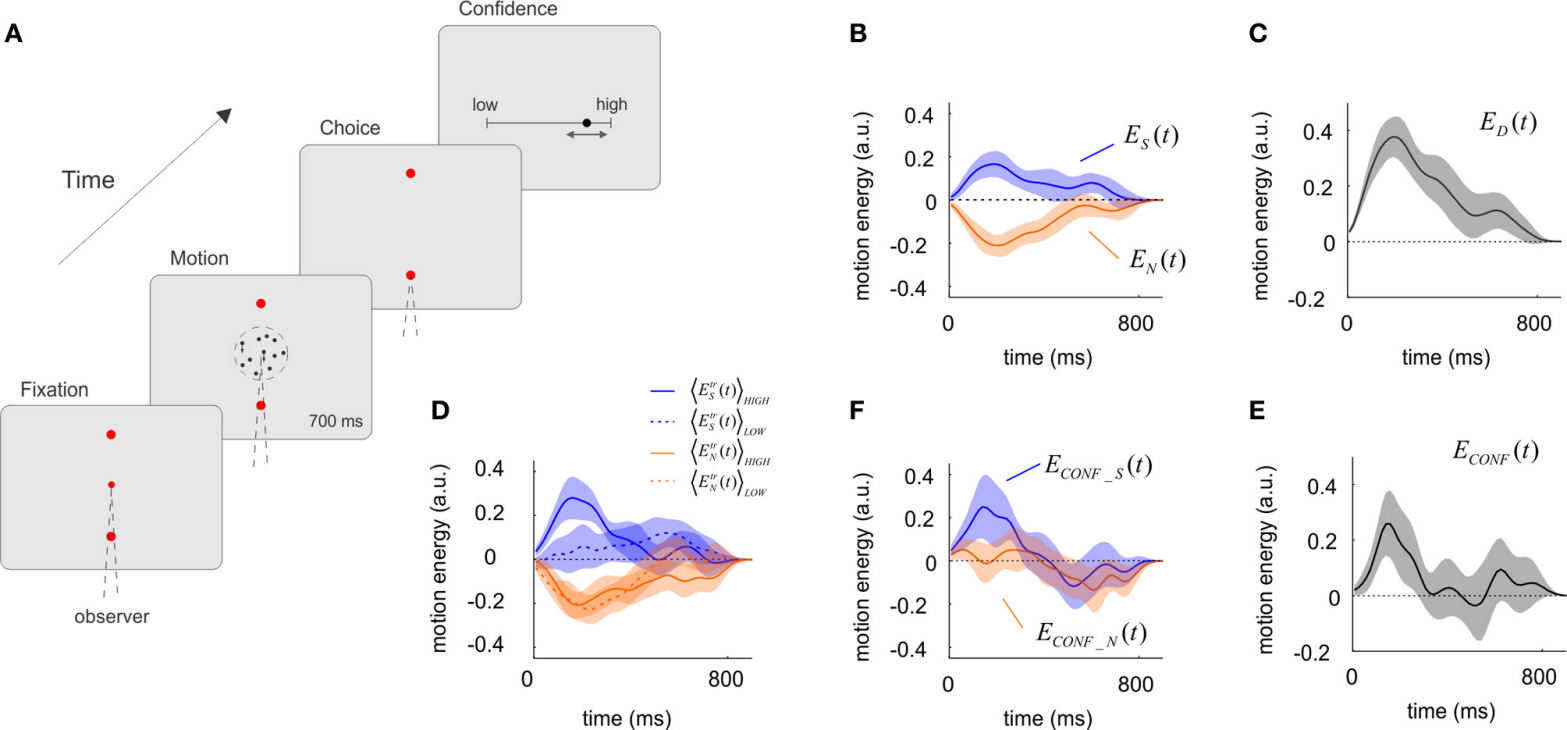
**The influence of sensory evidence on choice and confidence derived from a motion discrimination task. (A)** A trial of the motion task. The motion stimulus was presented for 700 ms after which participants signaled the response with an eye-movement and made a confidence report on a continuous scale ranging from complete chance on the left to absolute certainty on the right. Motion coherence was set to maintain accuracy at 67%. On average, confidence reports on correct trials were higher than on incorrect trials (one-tailed paired *t*-test, *p* < 5.10^−8^, *df* = 18). **(B)** Influence of motion energy fluctuations on choice. Motion energy fluctuations were obtained by applying a filter to the random dot stimulus and subtracting the mean motion energy set by the trial's motion coherence and direction. Upward and downward fluctuations were derived independently, and then averaged for the selected (blue) and non-selected (orange) directions. On average, fluctuations made motion energy in the selected direction to be above the mean, and motion energy in the opposite direction to be below the mean. **(C)** Time course of the decision kernel, obtained as the difference between *E*_*S*_(*t*) and *E*_*N*_(*t*). **(D)** Motion energy fluctuations for the selected and non-selected directions split according to confidence ratings into high (solid line) and low (dotted line) confidence. **(E)** Time course of the confidence kernel, showing that motion information had an early effect on confidence. **(F)** Motion energy fluctuations for the selected and non-selected directions, after subtracting low confidence trials from high confidence trials (shown in panel **D**). In panels **B–F**, shaded regions indicate SEM. Motion energy signals were smoothed with a Gaussian kernel with standard deviation of 40 ms.

Participants observed a random-dot kinematogram for a fixed duration of 700 ms. When the stimulus disappeared, they made an eye-movement toward a target in the direction of motion (upward or downward, Figure 1A). Following the eye movement (“choice”), participants indicated the degree to which they considered their response to be correct (“confidence”), reporting on a continuous scale going from chance to complete certainty. The number of coherently moving dots (called the “motion coherence”) was adjusted to maintain the proportion of correct choices at 67% (Watson and Pelli, 1983).

Applying a set of motion-sensitive filters following a procedure described by Adelson and Bergen (1985) we measured for each trial the momentary motion evidence in the upward and downward directions. These filters compute independently upward and downward motion (Adelson and Bergen, 1985); hence, two dots moving with equal speed in opposite directions will generate net and equal motion energy in both directions even if the average motion is zero.

In each trial, the random dot stimulus gives rise to motion information that varies in magnitude and direction. On average, the fluctuations in motion energy should cancel and motion energy should only reflect the mean motion coherence. However, at any moment in any particular trial, the stimulus contains motion information which influences the subjects' choice. Previous studies have shown that the time-varying contribution of motion information on choice can be recovered by reverse-correlation methods (Kiani et al., 2008; Resulaj et al., 2009). The main novelty of our study is the use of noise correlation analysis to the study of decision confidence.

From each trial (*tr*) we computed the fluctuations in motion energy in the selected *E*^*tr*^_*S*_(*t*) and the non-selected *E*^*tr*^_*N*_(*t*) directions. These quantities were computed so that they only reflect deviations from the mean set by the motion coherence and direction of motion. Motion energies were then averaged across trials and participants, to obtain the time-varying signals *E*_*S*_(*t*) = 〈E^*tr*^_*S*_(*t*)〉_*tr*_ and *E*_*N*_(*t*) = 〈E^*tr*^_*N*_(*t*)〉_*tr*_, which measure the average influence of motion in the selected and non-selected directions at different moments of the trial (Figure 1B). Comparison of *E*_*S*_(*t*) and *E*_*N*_(*t*) shows that the probability of choosing a direction of motion (i.e., upwards) may increase either because motion energy in the upwards direction was above the mean (positive *E*_*S*_(*t*)) or, alternatively, because motion energy in the opposite direction was below the mean [negative *E*_*N*_(*t*)] (Figure 1B). In fact, *E*_*N*_(*t*) is virtually the mirror image of *E*_*S*_(*t*) [i.e., *E*_*S*_(*t*) ~ −*E*_*N*_(*t*)] which was confirmed by a *t*-test comparing the areas under both curves (measured for each individual subjects) which showed no significant differences (*p* = 0.57, *t* = 0.58, *df* = 18) (Figure 1B). This result is expected since the formal solution of the task requires an estimation of the difference between upward and downward motion energy. In simple words, this indicates that positive votes in favor of a choice and the lack of votes in favor of the opposite choice contribute equally and add to form a decision. To combine these two measures we define the decision kernel as:

(1) $$E_{D}(t)=E_{S}(t)-E_{N}(t)$$

i.e., the difference between the fluctuations relative to the mean of the motion energy in the selected and the non-selected directions. *E*_*D*_(*t*) measure the influence of the combined motion signal on the perceptual choice at each moment in time (Figure 1C). As in previous studies (Kiani et al., 2008) we observed that the choice kernel in motion judgments peaks around 200 ms—but remained significantly above zero during the entire period of stimulus viewing (Figure 1C).

Our main objective was to investigate how sensory evidence contributes to subjective confidence. To this aim, we first categorized confidence reports, which were made on a continuous scale, in high- or low-confidence, using a median-split criterion applied to each individual session. Motion energies for the selected and non-selected directions were independently obtained for the high- and low-confidence trials (Figure 1D), and their difference calculated according to:

(2) $$E_{\text{CONF}\_S}(t)={\langle E_{S}^{tr}(t)\rangle }_{\text{HIGH}}-{\langle E_{S}^{tr}(t)\rangle }_{\text{LOW}}$$

(3) $$E_{\text{CONF}\_N}(t)={\langle E_{N}^{tr}(t)\rangle }_{\text{HIGH}}-{\langle E_{N}^{tr}(t)\rangle }_{\text{LOW}}$$

As in Equation (1), we combined these two signals according to:

(4) $$E_{\text{CONF}}(t)=E_{\text{CONF}\_S}(t)-E_{\text{CONF}\_N}(t),$$

which describes how the combined statistics of the noise affects confidence reports.

We then compared the influence of motion information on choice and confidence. The time-course of the confidence kernel [*E*_CONF_(*t*)] was significant over a narrower temporal window than the decision kernel [*E*_*D*_(*t*)] (Figure 1E). To quantify this effect, we computed the last time for which confidence and choice kernels were 1 s.e. above zero. The corresponding times were 0.26 ± 0.09 s for confidence, and 0.73 ± 0.12 s for choice (mean and standard errors were derived from Jackknife estimates based on individual sessions). A *t*-test revealed that the difference between these latencies was significant (*t* = 2.96, *p* < 0.005, *df* = 37). These results show that early moments of the decision have a net effect on confidence judgments.

The most interesting observation arises when confidence is analyzed separately for motion in the selected *E*_CONF_*S*_(*t*) and non-selected *E*_CONF_*N*_(*t*) directions. We observed an asymmetrical dependence revealing that *E*_CONF_*S*_(*t*) (i.e., fluctuations of motion in the selected direction) had a higher effect on confidence than *E*_CONF_*N*_(*t*) (Figure 1F). To test the significance of this observation, we measured for each individual subject the difference in the area under each curve, computing the quantity $\left(\int_{0}^{T}E_{\text{CONF}\_S}(\tau )-E_{\text{CONF}\_N}(\tau )\times (-1)d\tau \right)$ for *T* = 300 ms. A *t*-test confirmed that this difference was significant (*P* < 0.05, *t* = 1.84, *df* = 18). This implies that when comparing high and low confidence trials, positive fluctuations in favor of the selected choice had a greater impact than negative fluctuations in the non-selected choice. We conducted a linear regression analysis to verify the early and asymmetrical impact of motion fluctuations on confidence judgments. For each session, the raw confidence data was sorted into deciles. Motion energy fluctuations toward and away from the selected target were averaged in an early (*T* < 0.3 s) and a late (*T* > 0.3 s) temporal window, and used (together with an intercept term) as independent variables to fit the confidence deciles. We found that only early motion fluctuations in selected direction had a significant effect on confidence (*p* < 0.005), consistent with our previous analysis.

### Experiment 2: choice and confidence in an RT task of luminance discrimination

**Figure 2:**
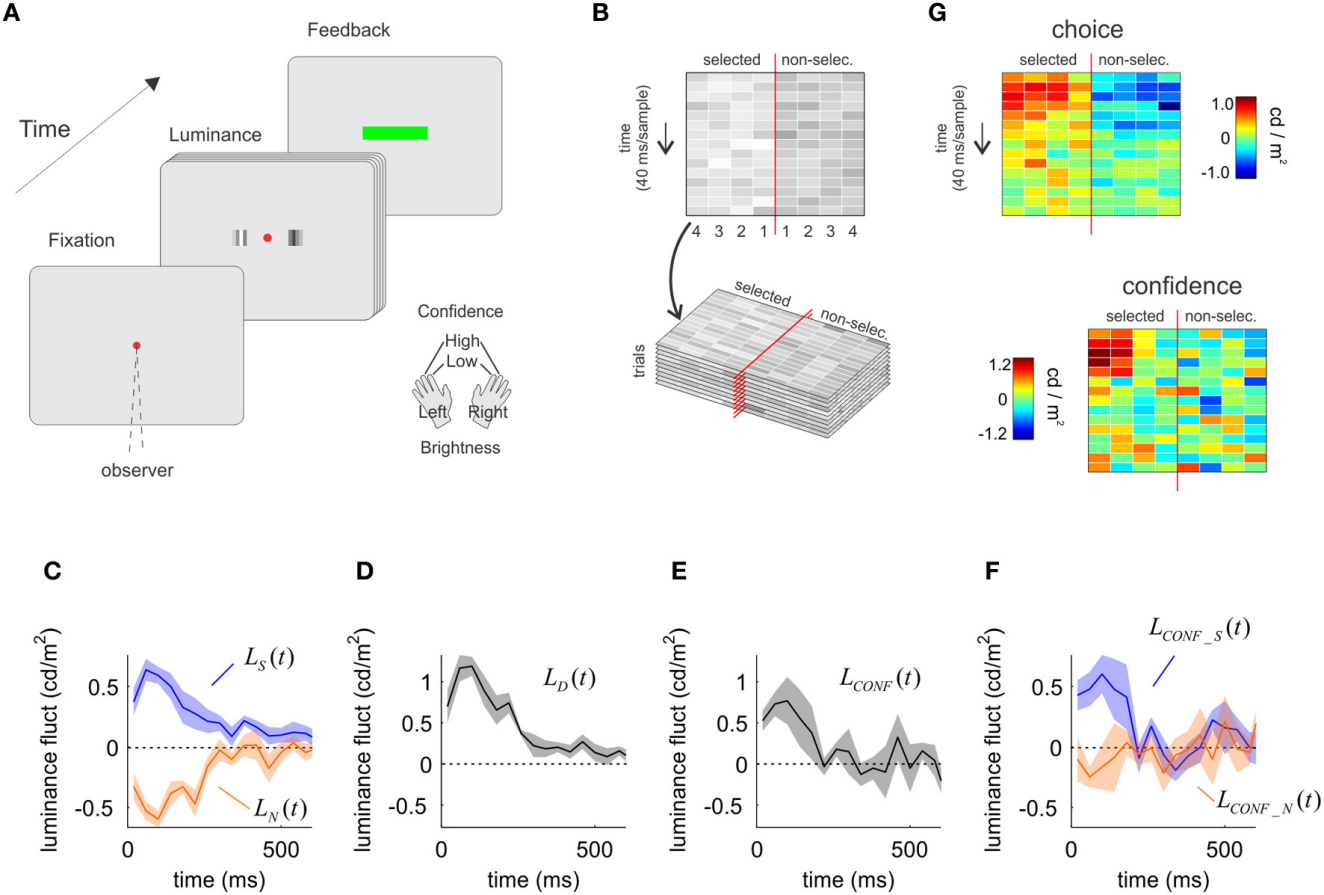
**The influence of sensory evidence on choice and confidence derived from a luminance discrimination task. (A)** A trial of the luminance task. Two patches of flickering bars (updated at 25 Hz) were presented until participants made a response. Participants indicated which patch is brighter and the confidence in their decision with a single manual response. **(B)** Spatiotemporal profile of the luminance signal. The red vertical line represents the fixation point, and the four columns to each side indicate the luminance in time of the four bars in each patch, numbered from the fovea to the periphery. **(C)** Time course of the influence of luminance fluctuations on choice. Luminance fluctuations were obtained by subtracting from the luminance of each bar the mean of the distribution used to sample the luminance values, and averaging across the four bars in each patch. On average, fluctuations were positive for the patch selected as the brighter (shown in blue), and negative (dimmer) for the opposite patch (orange). **(D)** Time course of the decision kernel, obtained as the difference between *L*_*S*_(*t*) and *L*_*N*_(*t*). **(E)** Time course of the confidence kernel, showing an early effect of luminance information on confidence decisions. **(F)** The average of the luminance fluctuations in low confidence trials was subtracted from that of high confidence trials, independently for the patch that was selected as the brighter (blue) and for the opposite patch (orange). The selected and the non-selected patches made unequal contributions to decision confidence. **(G)** Same as in panels **C** and **F**, but preserving the spatial structure of the stimulus (i.e., not averaging across the four bars in each patch before computing the decision and confidence kernels). In panels **C**–**F**, shaded regions indicate SEM.

A potential confound of the motion discrimination task is that momentary motion energy in both directions is partially correlated (at least in the standard version of the task in which the number of points is fixed as we used here). Thus, the two signals used to investigate the time-course of the construction of confidence are not fully independent. To discard this confound and test the robustness of our findings, we performed a second independent experiment in which participants decided which of two patches, located at opposite sides of a fixation point, was brighter. Each patch was composed of four vertical, spatially adjacent bars (Figure 2A). The luminance of each bar was resampled independently every 40 ms from a Gaussian distribution with equal variance. The mean luminance was set higher for one of the two patches (“target”), adjusted to keep the proportion of correct responses at 75% (Watson and Pelli, 1983). Since the goal of this experiment was to test the generality of our findings we changed several additional parameters, detailed in the “Materials and Methods” section which we reasoned could constitute potential confounds. In particular, stimulus viewing time was not fixed and participants were free to decide when to make a response.

The analysis follows exactly the same procedure as in the motion task, measuring the influence of noise fluctuations on choice and confidence through psychophysical reverse-correlation methods. We measured luminance fluctuations averaged across all trials for the selected and non-selected patches. This was done independently for each of the four bars of the patch, to obtain *L*^*b*^_*S*_(*t*) and *L*^*b*^_*N*_(*t*), where the super index *b* ranges from 1 to 4 and denotes the position of the bar from the fovea to the periphery (Figure 2B). Then, we averaged across all spatial locations: *L*_*S*_(*t*) = 〈L^*b*^_*S*_(*t*)〉_*b*_ and *L*_*N*_(*t*) = 〈L^*b*^_*N*_(*t*)〉_*b*_ to measure the temporal course of luminance fluctuations for the selected and non-selected patches.

Momentary luminance in the selected [*L*_*S*_(*t*)] and non-selected [*L*_*N*_(*t*)] patches had comparable influences on choice (Figure 2C) (testing whether the area of the selected and non-selected patches were different over the first 300 ms was not significant, *P* = 0.381, *t* = 0.98, *df* = 4). As with the motion task, this result is expected since the formal solution of this task requires an estimation of the difference of luminance between the patches. For instance, participants may opt for the right patch either because it is bright (positive votes favoring right) or because the left patch is dim (absence of votes favoring left).

We combined momentary luminance fluctuations in the selected and non-selected patches to compute the luminance decision kernel *L*_*D*_(*t*) = *L*_*S*_(*t*) − *L*_*N*_(*t*). The luminance decision kernel peaked on the third sample (centered at 100 ms) (Figure 2D) in consistency with previous reports (Ludwig et al., 2005).

As for the motion experiment, we measured the difference between fluctuations of luminance obtained from high and low confidence trials, for the selected and non-selected patch:

(5) $$L_{\text{CONF}\_S}^{b}(t)={\langle L_{S}^{b,\;tr}(t)\rangle }_{\text{HIGH}}-{\langle L_{S}^{b,\;tr}(t)\rangle }_{\text{LOW}}$$

(6) $$L_{\text{CONF}\_N}^{b}(t)={\langle L_{N}^{b,\;tr}(t)\rangle }_{\text{HIGH}}-{\langle L_{N}^{b,\;tr}(t)\rangle }_{\text{LOW}}$$

We averaged across the four bars in each patch to obtain: *L*_CONF_*S*_(*t*) = 〈*L*^*b*^_CONF_*S*_(*t*)〉_*b*_ and *L*_CONF_*N*_(*t*) = 〈*L*^*b*^_CONF_*N*_(*t*)〉_*b*_ and, as in Equation (4), combined these two signals to derive the luminance confidence kernel:

(7) $$L_{\text{CONF}}(t)=L_{\text{CONF}\_S}(t)-L_{\text{CONF}\_N}(t)$$

As for motion, the time-course of the luminance confidence kernel was significant over a narrower temporal window than the choice kernel (Figure 2E). The last times for which the choice and confidence kernels were significant (1 s.e. above zero for three consecutive samples) were 0.19 ± 0.05 s for confidence, and 0.73 ± 0.21 s for choice (mean and standard errors were derived from the Jackknife estimates based on individual sessions). A *t-test* revealed that the difference between these measures was significant (*t* statistics, *P* < 0.05, *t* = 2.76, *df* = 9).

When confidence was analyzed separately for luminance in the selected [*L*_CONF_*S*_(*t*)] and non-selected [*L*_CONF_*N*_(*t*)] patches, we observed an asymmetrical dependence revealing that mainly luminance fluctuations in the selected patch affected confidence (Figure 2F). A *t*-test confirmed that momentary luminance in the selected and non-selected patch had a different influence on confidence (*P* < 0.05, *t* = 2.99, *df* = 4).

Repeating the exact same analysis independently for each bar of the patch (instead of collapsing them in the average luminance for each patch) shows the robustness of this analysis (Figure 2G). The spatiotemporal profile of influences of luminance on choice shows a very similar pattern for the selected and non-selected patches (with opposite signs). For confidence, there is a clustered influence of the early samples of the selected patch with is biased toward the periphery, while the spatiotemporal influence of the non-selected patch shows only a very noisy and unstructured distribution (see Discussion).

## Psychophysical kernels predicted by current models of choice and confidence

The motion and luminance experiments revealed highly consistent patterns despite involving different visual attributes (luminance and motion), response modality (fix time or freely responding), response effectors (eye movement or manual responses), and response order (two consecutive responses indexing choice and confidence independently or a single response grouping choice and confidence). Our results show that in both cases, noise fluctuations had a relatively homogeneous effect on choice. Using the vote analogy, participants may choose an option either because it had many votes or because the opposite option did not have any favoring votes (the other patch was dim, the opposite motion direction had no energy). This information is accumulated during a broad temporal window. Confidence acts as a rectifier, mainly weighting votes in favor of the selected choice.

The specific blindness of the confidence system, which has access to a distorted readout of the sensory signal, was surprising and intriguing. Since we showed that it was robust and replicable to different setups we reasoned that it might derive from basic aspects of the decision making machinery. Next, we explore whether our findings can be accounted by current standard models of confidence and decision-making.

The decision process has been modeled as a noisy integrator that accumulates evidence provided by the sensory systems (Vickers, 1970; Gold and Shadlen, 2007; Ratcliff and McKoon, 2008). Two canonical alternatives of binary decisions have been proposed to describe how perceptual evidence is stochastically accumulated in time until a threshold: the “race” and the “random-walk” models. In the race model each signal is integrated independently until the first hits a decision boundary. In the random-walk model, it is the difference between both signals (instead of each of them independently) that is integrated to a threshold. These two modules can be seen as a continuum (Bogacz, 2007; Moreno-Bote, 2010) in which the parameter which weights the cross-talk between both tasks is varied. When it is set to zero, the decision is based on a race. When it is set to 1 the model is based on a random-walk. Intermediate values of cross-talk lead to hybrid models which integrate in parallel evidence in favor of both decisions while also conveying information about the difference between both signals.

We simulated different variants of competing-accumulation models in a task which required the accumulation of time-varying sensory evidence, and fitted the models to the data obtained from the luminance task. For each model, the state of the two accumulators is described by two variables L and R, which count votes in favor of the left and right responses, respectively, and are updated according to:

(8) $$L_{i+1}=L_{i}+\left(\mu \times s+\eta_{L,\;i}-\rho (\eta_{R,\;i}+\mu \times (1-s))\right)$$

and

(9) $$R_{i+1}=R_{i}+\left(\mu \times (1-s)+\;\eta_{R,\;i}-\rho (\eta_{L,\;i}+\mu \times s)\right)$$

**Figure 3:**
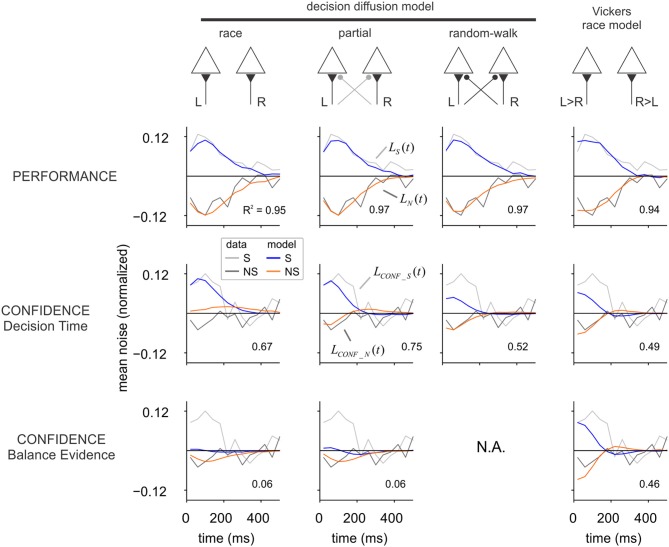
**Alternative models of decision and confidence.** Each column shows the performance of a different model, sketched in the upper part of the figure. In the race model each signal (L and R) is integrated independently until the first hits a decision boundary. In the random-walk model, it is the difference between both signals (instead of each of them independently) that is integrated to a threshold. In the partial model, evidence for the opposite alternative is integrated but less efficiently. In Vickers race model, the sign of the difference between L and R defines which of the two accumulators is updated in each time step. For each model, we computed the influence of fluctuations on choice and confidence. In the upper row (“Performance”), the average of the input noise to the winning (S) and losing (NS) accumulators is shown in blue and orange, respectively. Gray and black curves show the noise averages from the luminance experiment (repeated from Figure 2C), which were used for fitting the models. Confidence was modeled according to two different criteria: confidence as a function of decision time, or confidence as a function of the “balance of evidence” (difference in the state of the accumulators at the moment of choice). The two rows in the bottom show the impact of input fluctuations on confidence. As for the experiments, the average of the input fluctuations in low confidence trials was subtracted from that of high confidence trials, independently for the winning (blue) and losing (orange) accumulator. A single parameter was used to fit the confidence kernels of the luminance task, which are shown in Figure 2F and repeated here in light (S) and dark (NS) gray. For both experiment and model, the amplitude of the kernels was normalized to the standard deviation of the noise. Models were fit to maximize the *R*-squared, which is indicated inside each graph.

In Equations (8) and (9), *i* is the time step, which is set to 40 ms to match the update rate of the luminance experiment. μ (the first free parameter of the model) is the mean signal difference between target and distractor. η_*L*_ and η_*R*_ are the noise in the left and right patches, respectively, sampled independently at each time step from a standard Gaussian distribution. *s* indicates whether the target patch is on the left (*s* = 1) or on the right (*s* = 0). The parameter ρ controls the degree of anti-correlation between the two accumulators. We explored three instantiations of this set of equations: ρ = 0 (*race* model), ρ = 1 (*random-walk* model), and ρ = 0.5 (*partial* model) (Figure 3).

The fourth model (last column in Figure 3) is a variant of the race model, inspired by the one adopted by Vickers in his models of confidence (Vickers, 1979), and thus referred here as Vickers race model. At each time step, the difference between the right and left signals was computed, and the only accumulator that was updated was the one for which the difference was positive. Specifically, at each time step we computed the difference: *d*_*i*_ = μ × s + η_*L,i*_ − (η_*R,i*_ + μ × (1 − *s*)), where the different variables are defined as in Equations (8) and (9). The accumulators were then updated as follows. If *d*_*i*_ > 0, then *L*_*i*+1_ = *L*_*i*_ + *d*_*i*_ and *R*_*i*+1_ = *R*_*i*_; else, if *d*_*i*_ < 0, *R*_*i*+1_ = *R*_*i*_ + *d*_*i*_ and *L*_*i*+1_ = *L*_*i*_.

For every model, the decision process continues until one of the accumulators reaches a threshold which signals the selected choice. The threshold level (*thres*) constitutes the second free parameter of each model. In the simulations, we considered that the decision variables L and R could only take positive values (which gives rise to the non-monotonic shape of the performance kernels). Decision kernels were computed as in the experiments, with η_*L*_ and η_*R*_ as the noise signals used to derive the choice and confidence kernels. Parameters μ and *thres* were adjusted to maximize the fraction of explained variance (see “Materials and Methods”). Independent fits were conducted for each model, based on 100,000 simulated trials. All models were capable of adequately fitting the decision kernels, as shown in Figure 3 (upper row).

After the optimal parameters were obtained fitting the decision kernels, we computed the probability of a high confidence report according to two commonly used criteria: (1) confidence as a function of decision time (the time step at which one of the accumulators reached the threshold) (Audley, 1960; Kiani and Shadlen, 2009), and (2) confidence as a function of the “balance of evidence” (the absolute difference in the state of the two accumulators, *BE* = *abs*(*L* − *R*), at the moment of choice) (Vickers, 1979).

To investigate which of these two mechanisms could account for confidence distributions, the probability of a high confidence trial was computed independently as a function of either *DT* (decision time) or *BE* (balance of evidence), according to a sigmoid function with only one parameter: *p*_+_ = (1+ exp (−*a*(*x* − *m*)))^−1^, where *p*_+_ is the probability of a high confidence response, *x* is either *BE* or *DT* across trials, *m* is the median of *x*, and *a* controls the slope of the sigmoid function.

For each model, the parameter *a* was fitted to the confidence kernels, maximizing the fraction of explained variance (see “Materials and Methods”). The analysis of the best-fitting models (Figure 3) shows that confidence kernels based on the balance of evidence hypothesis predict either a symmetrical effect of fluctuations in the selected and unselected alternatives (Vickers's race model), or predict that the fluctuations from the unselected alternative would have a larger effect than those at the selected one, exactly the opposite of what we found in the motion and luminance experiments. The larger impact of the fluctuations from the unselected side is expected under the balance of evidence hypothesis, since the accumulator for the selected choice is always at the threshold when a decision is made and thus most of the variability in the balance of evidence depends on the state of the losing accumulator. The small effect of the fluctuations in the selected patch (initially positive, then negative), can be explained by the fact that fast responses will tend to be associated with higher confidence because the accumulator corresponding to the non-selected alternative has less time to drift closer to the threshold. As previously noted (Vickers, 1979), the balance of evidence hypothesis is incompatible with the random-walk model since each accumulator is the mirror image of the other and thus the difference is constant at the moment of choice (for thresholds that do not vary with time).

The analysis of the confidence kernels based on decision time shows that Vickers's race model and the random-walk model both predict that sensory evidence from the selected and non-selected alternatives would make equal contributions to the confidence judgment. The alternative race model—where each accumulator integrates evidence from a single spatial location—does predict a reduced influence of evidence from the non-selected patch, but the sign of this influence is opposite to the one observed in the experiment. A model with partial interaction between both accumulators, which stands between the race and random-walk models (the *partial* model with ρ = 0.5), can simultaneously describe the main qualitative findings for both the decision and confidence kernels (Figure 3).

## Discussion

Relying on an extension of psychophysical reverse correlation methods methods (Ahumada, Jr., 1996; Gosselin and Schyns, 2001), we derived the temporal kernels describing how sensory information influences choice and confidence judgments. Our results show that: (1) confidence is mostly influenced by the initial moments of the decision and (2) confidence is sensitive to evidence for the selected choice but is virtually blind to evidence against the non-selected choice. These results were consistently observed in two independent experiments. Simply to avoid misinterpretations we also emphasize that our results are agnostic relative to when confidence is being made (Baranski and Petrusic, 1998); they indicate which subset of the signal contributes to confidence but make no claim on whether this information is used in real-time or retrospectively accessing a distorted memory of the sensory signal.

The early influence of sensory evidence on confidence seems incompatible with recent models that postulate that when confidence reports are required, sensory evidence continues to accumulate after the commitment to a choice (Pleskac and Busemeyer, 2010). These models would predict temporal kernels for confidence which remain significant after the choice kernels have faded out.

The observation that confidence is influenced mainly by evidence in favor of the selected choice is also incompatible with random-walk models of decision-making, a popular mathematical model where two-alternative decisions are described by a one-dimensional system that integrates over time the difference between two noisy inputs, such that a choice is made when it reaches a threshold (Ratcliff and McKoon, 2008). In these models, sensory evidence in favor of a decision automatically translates into evidence against the other (Kiani and Shadlen, 2009), which makes them unable to explain the asymmetric influence of different pieces of sensory evidence. Our results show that to account for these observations, distortions in this conversion process ought to be incorporated.

Interestingly, these distortions in the accumulation of evidence occur naturally in neurophysiological models of decision-making based on attractor networks (Usher and McClelland, 2001; Wang, 2002; Wong and Wang, 2006). These models rely on the competitive interaction of pools of neurons representing alternative decisions, interacting through lateral inhibition and self-excitation. As in random-walk models, each accumulator is affected by sensory inputs bearing on both alternatives. Crucially, the influence of different sensory inputs is subjected to different latencies, since lateral connections are slow. These slow connections are required to attain stable firing rates in both spontaneous and memory regimes (Brunel and Wang, 2001), implement winner-take-all dynamics, and model the slow ramping activity observed in LIP in the random-dot motion task (Wang, 2002). Thus, it is conceivable that the distortion in the accumulation process required by our best-fitting model may be due to delays introduced by slow lateral connections in attractor networks.

Most models of confidence judgments based on independent accumulators relied on Vickers's “balance of evidence” hypothesis, which postulate that confidence is a function of the difference in accumulated evidence between both accumulators once one of them reaches the decision threshold (Vickers, 1979). Assuming a fixed response threshold, this readout predicts either symmetrical kernels of confidence, or confidence kernels with a lower influence from the selected alternative. These results are opposite to our behavioral findings. However, with the simple *ad-hoc* assumption that only the evidence supporting the selected integrator is accessible by the meta-cognitive system, the asymmetry can be easily recovered. Partial support for this assumption results from the observation of a limited-capacity readout of internal variables in different experimental setups. For instance, in a previous study we demonstrated that introspection of response time is tightly correlated with objective response time in a single-task context. However, in a dual task paradigm, the objective processing delay resulting from interference by a second concurrent task was totally absent from introspective estimates (Corallo et al., 2008; Marti et al., 2010). In another dual-task psychophysical experiment, it was shown that a categorical choice on the direction of motion conditions which subset of the sensory information is used subsequently to make fine direction discrimination (Jazayeri and Movshon, 2007). Similarly, in our experiment, it is then reasonable to assume that a choice made in a binary decision may alter the subset of sensory information available for a subsequent confidence judgment. Under this view, even if the early samples are the most decisive ones, information for the confidence system is read retrospectively and top-down mechanisms to retrieve this information may selectively weight information in circuits encoding the selected choice.

Evidence in favor of a limited-capacity of the meta-cognitive readout of sensory system also comes from an analysis of the contribution of each individual bar in the patch to choice and confidence (Figure 2G). Consistently with our observations in the temporal domain, only a subset of the sensory information used for choice is accessible for meta-cognitive judgments. Also, the more peripheral bars of the patch had a stronger influence on confidence. This result may seem surprising but is compatible with a view in which the movement of attention inflicted by a spatial choice biases the weights of sensory evidence. The mechanistic explanation of this process should be a matter of further investigation; in this article we concentrated on understanding the temporal construction of choice and confidence judgments.

Our results support theories of confidence as a function of decision time (Audley, 1960). In other words, for an external observer, knowing the decision time should be sufficient to determine confidence. It is tempting to assume that our brains proceed in the same way, mapping estimation of confidence to estimation of time. In fact, elapsed time can be computed while a decision is being made simply estimating the area under the ramping curve. This is a very simple geometric argument when evidence is accumulated to a fixed threshold: if the height is fixed, the area of a triangle determines its base. Hence, the estimation of time and therefore of confidence, may rely on an integration of activity of neurons which in turn integrate sensory evidence. This process would literally instantiate the notion that confidence judgments results from a decision about a decision, i.e., a hierarchical cascade of canonical circuits implementing decisions with different levels of abstraction relative to the external world (McClelland, 1979).

## Materials and methods

### Participants

Nineteen participants took part in the motion experiment. Each participant performed 2 sessions of 600 trials each, in blocks of 100 trials. Five participants took part in the luminance experiment. Each participant performed 2 sessions of 480 trials each, in blocks of 160 trials. All participants were college students, aged between 20 and 30 years old. The study was approved by a local ethical committee for biomedical research, and informed consent was obtained from all subjects.

### Motion

Stimuli were generated following the procedures described previously by Cook and Maunsell (Cook and Maunsell, 2004). The random dots were white, presented on a black background, had a diameter of 0.14° and appeared within a 6.5° circular aperture centered on the fixation point. White dots were grouped in two patches, each updated every other frame (frame rate = 60 Hz) using the following procedure. The dots of one patch were replaced with a new set of randomly positioned dots. Dots in the other patch were displaced by a fixed distance of 0.33°. The dots in the later patch determined the coherence of motion. For 0% coherence, each dot moved by the same amount, in a random direction. For 100% coherence, they moved the same fixed distance, all in the same direction. On the next update, the groups were switched. This procedure assured that participants cannot obtain motion information by tracking individual dots. The motion coherence displayed on each trial was adjusted online to keep the proportion of correct responses at 67%, with a Quest procedure (Watson and Pelli, 1983). Motion coherence was corrupted with additive noise sampled from a Gaussian distribution with a mean of zero and a standard deviation of 7% (in coherence scale), resampled every 4 frames. Each trial started with the presentation of a red fixation dot (diameter of 0.2°) on a black background which remained visible. The motion stimulus was presented after the participant had fixated on a central red fixation point for 1000 ms. Gaze position was monitored with an EyeLink 1000 eyetracker, at a sampling frequency of 1000 Hz, and a response was computed when the gaze was within 2.5° of one of the two targets. The experiment was programmed using the Psychophysics Toolbox extensions for Matlab (Mathworks) (Pelli, 1997).

### Luminance

Participants fixated a central red dot (diameter of 0.56°) on a gray background (50 cd/m^2^) for 200 ms. Two flickering gray patches were presented at both sides of the fixation dot until a response was made. Patches were presented on the horizontal meridian, centered at ±1.04° from the fixation point. Each patch was composed of four vertical, spatially adjacent bars (0.14° × 0.56°). The luminance of the bars was updated synchronously every 40 ms, sampling from a Gaussian distribution with a standard deviation of 10 cd/m^2^. The mean of this distribution equaled the luminance of the background for one of the patches and was set higher for the other (referred as “target”). Trials in which participants selected the target were considered correct. The mean luminance of the target was adjusted to keep the proportion of correct responses at 75% (Watson and Pelli, 1983). Responses were informed through the keyboard: participants pressed keys *A* or *S* to indicate that the brighter patch was on the left (respectively, corresponding to a low and high confidence choice) and keys *K* or *L* to indicate that the brighter patch was on the right (respectively, corresponding to a low and high confidence choice). After each response, a green or red rectangle indicated whether the choice was (respectively) correct or incorrect. The experiment was programmed using Cogent 2000, as implemented in Matlab (Mathworks).

### Motion energy

To quantify the fluctuations in motion during the course of each trial, we filtered the sequence of random dots with spatiotemporally oriented filters following the procedure described in previous studies (Adelson and Bergen, 1985; Kiani et al., 2008). Spatiotemporally oriented filters were constructed by adding the outputs of two separable filters. The spatial impulse response functions were defined as:

$$\begin{array}{c} \text{even}(x,\;y)=\cos(2\pi \alpha y)\exp\left(-\frac{y^{2}}{\sigma_{y}^{2}}\right)\exp\left(-\frac{x^{2}}{\sigma_{x}^{2}}\right)\\ \text{odd}(x,\;y)=\sin\left(2\pi \alpha y\right)\exp\left(-\frac{y^{2}}{\sigma_{y}^{2}}\right)\exp\left(-\frac{x^{2}}{\sigma_{x}^{2}}\right), \end{array}$$

and the temporal impulse response functions as:

$$\begin{array}{c} \text{fast}(t)={\left(kt\right)}^{3}\exp(-kt)\left[\frac{1}{3!}-\beta \frac{{\left(kt\right)}^{2}}{(3+2)!}\right]\\ \text{slow}(t)={\left(kt\right)}^{5}\exp(-kt)\left[\frac{1}{5!}-\beta \frac{{\left(kt\right)}^{2}}{(5+2)!}\right], \end{array}$$

with α = 0.56, *k* = 100s^−1^, σ_*y*_ = 0.97°, σ_*x*_ = 0.02° and β = 0.9.

The two spatial and two temporal responses can be combined into four separable spatiotemporal responses. Motion filters were constructed by combining linearly these spatiotemporal responses, resulting in two upwards- and two downwards- selective filters (Adelson and Bergen, 1985; Kiani et al., 2008). The filters were then convolved with the motion dot pattern, after binning the dots presented on each frame in a spatial grid of 100 × 100 bins covering the whole stimulus. The output of the two filters selective to the same direction of motion were squared and summed. The two resulting signals provide a good estimate of the motion energy in the upwards- and downwards- directions at each point in the image and as a function of time.

Since our aim was to understand the temporal course of choice and confidence, we summed the energies across space for each individual trial. The mean motion energy was removed from each trial, as we were interested in the impact of the motion fluctuations deviating from the trial's mean. The mean motion energy was estimated independently for each direction of motion (toward or away from the target) and time frame, fitting a linear regression model with motion coherence and intercept as independent variables and motion energy as dependent variable. After subtraction of the mean, we obtained two signals from each trial quantifying the motion energy fluctuations in the upward and downward directions as a function of time.

### Model fitting

We compared our results to four alternative models of decision and confidence. Each model contained three parameters (μ, *thres*, and *a*). The parameters were adjusted to maximize the *R*-squared statistic:

$$R^{2}=1-\frac{SS_{\text{MODEL}}}{SS_{\text{DATA}}},$$

where

$$\begin{array}{l} SS_{\text{MODEL}}=[\sum_{t<320\text{ ms}}{\left(X_{S}^{\text{DATA}}(t)-X_{S}^{\text{MODEL}}(t)\right)}^{2}+\sum_{t<320\text{ ms}}{\left(X_{\text{NS}}^{\text{DATA}}(t)-X_{\text{NS}}^{\text{MODEL}}(t)\right)}^{2}]\\ SS_{\text{DATA}}\;=\left[\sum_{t<320\text{ ms}}{\left(X_{S}^{DATA}(t)\right)}^{2}+\sum_{t<320\text{ms}}{\left(X_{\text{NS}}^{\text{DATA}}(t)\right)}^{2}\right] \end{array}$$

*S* stands for “selected” side, NS for “non-selected” patch, and *X* is the average of the noise in the conditions specified by the indexes (corresponding to the data points shown in Figure 3).

### Conflict of interest statement

The authors declare that the research was conducted in the absence of any commercial or financial relationships that could be construed as a potential conflict of interest.
